# Selecting long-term care facilities with high use of acute hospitalisations: issues and options

**DOI:** 10.1186/1471-2288-14-93

**Published:** 2014-07-22

**Authors:** Joanna B Broad, Toni Ashton, Thomas Lumley, Michal Boyd, Ngaire Kerse, Martin J Connolly

**Affiliations:** 1 Freemasons’ Department of Geriatric Medicine, University of Auckland, C/- WDHB, Box 93503, Takapuna, Auckland 0740, New Zealand; 2 School of Population Health, University of Auckland, Auckland, New Zealand; 3 Department of Statistics, University of Auckland, Auckland, New Zealand; 4 School of Nursing, University of Auckland, Auckland, New Zealand; 5 Health of Older People, Waitemata District Health Board, Auckland, New Zealand

**Keywords:** Long-term care, Risk assessment, Hospitalization, Health services for the aged, facility selection, Research design

## Abstract

**Background:**

This paper considers approaches to the question “Which long-term care facilities have residents with high use of acute hospitalisations?” It compares four methods of identifying long-term care facilities with high use of acute hospitalisations by demonstrating four selection methods, identifies key factors to be resolved when deciding which methods to employ, and discusses their appropriateness for different research questions.

**Methods:**

OPAL was a census-type survey of aged care facilities and residents in Auckland, New Zealand, in 2008. It collected information about facility management and resident demographics, needs and care. Survey records (149 aged care facilities, 6271 residents) were linked to hospital and mortality records routinely assembled by health authorities. The main ranking endpoint was acute hospitalisations for diagnoses that were classified as potentially avoidable. Facilities were ranked using 1) simple event counts per person, 2) event rates per year of resident follow-up, 3) statistical model of rates using four predictors, and 4) change in ranks between methods 2) and 3). A generalized mixed model was used for Method 3 to handle the clustered nature of the data.

**Results:**

3048 potentially avoidable hospitalisations were observed during 22 months’ follow-up. The same “top ten” facilities were selected by Methods 1 and 2. The statistical model (Method 3), predicting rates from resident and facility characteristics, ranked facilities differently than these two simple methods. The change-in-ranks method identified a very different set of “top ten” facilities. All methods showed a continuum of use, with no clear distinction between facilities with higher use.

**Conclusion:**

Choice of selection method should depend upon the purpose of selection. To monitor performance during a period of change, a recent simple rate, count per resident, or even count per bed, may suffice. To find high–use facilities regardless of resident needs, recent history of admissions is highly predictive. To target a few high-use facilities that have high rates after considering facility and resident characteristics, model residuals or a large increase in rank may be preferable.

## Background

The question “Which long-term care facilities have residents with high use of acute hospitalisations?” may seem straightforward, though may be asked for many purposes. This study arose when our health research group wished to recruit high-use facilities to test an intervention aiming to reduce hospitalisations by improving care within facilities. But other researchers may wish to select a few facilities with high rates of acute admissions in order to test a resident-specific intervention. In other contexts a manager of a hospital clinical outreach programme may want to provide better supports and services to residential long-term care (LTC) facility staff, to avert resident admissions to acute hospital care. An auditing authority may wish to include avoidable hospital presentations in its monitoring of facility performance. In general, such questions frequently relate to an interest in reducing admissions through trials or service interventions, and this is the main justification of this paper, but questions may also regard admissions as a marker of care quality or have other purposes.

Although some admissions are needed for good care, reducing acute admissions from LTC appears well justified. Better preventive care and emphasis on managing acute illness in situ may improve resident health outcomes [1,2]. Older adults hospitalized acutely can be harmed; some decline in a few days, and iatrogenic complications of acute illness may occur because of a hospital’s more aggressive interventions, less attention to mobility and nutritional needs, and exposure to infections [3-7]. Care transitions are more disruptive for older people, particularly those with dementia [8]. Acute hospitalisations are often associated with negative impacts at the end-of-life [9]. From an economic perspective, hospital care costs more than LTC, so where outcomes are similar or better for a given condition, funds may be better used elsewhere [10,11].

Acute hospital admissions from LTC are of wide importance as LTC is often used in later life. In Auckland (population 1.3 million), 28% of those aged 85+ years live in LTC [12]. In other countries, LTC residents have twice the rate of acute hospitalisations and longer age/sex adjusted acute length of stay than non-residents [13-15]. In OPAL, a census-type survey of all residents in LTC conducted in Auckland in 2008, 4% of all LTC residents were referred to a hospital emergency department (ED) in the two weeks prior to the survey. However, there are no routine reports of hospital presentations, hospital admissions, bed-days or costs of LTC residents available for NZ.

The research question – how to identify facilities with high use – arose for these investigators when planning the Aged Residential Care Healthcare Utilization Study (ARCHUS), a randomized controlled trial of a complex, multi-disciplinary facility-level intervention intended to reduce avoidable acute hospitalisations conducted in Auckland [16]. The study design required that before commencing recruitment, high-use facilities be identified in order to enrol those with greatest chance of demonstrating a change in resident outcomes. (Separate modelling was undertaken to identify characteristics of residents and facilities to inform care model development). Selection methods that were discussed early on ranged from simple rates of hospital admissions per facility bed over a defined period, to those that attempted to adjust for facility characteristics and resident need levels in complex statistical models.

To identify high-use facilities in situations where suitable data are not readily available but neither are research funds for the purpose, the question becomes “If reliant on existing data, which of many possible selection methods is most appropriate?” In this reflective paper we consider some issues and options that may be relevant when responding to the question, we form these into a framework, use the ARCHUS study to demonstrate four selection methods and show the variation in selections they make, and discuss when methods may be more appropriate than others. The paper intends to focus more on the approach taken rather than provide specific results which we consider are unlikely to be of great relevance to others not in our particular situation.

## Methods

### Aspects of selection

During the ARCHUS trial planning, much discussion revolved around methods of addressing the question of facility selection, with a large number of aspects or measures that could be relevant, of residents and of facilities. In choosing the method for ARCHUS, several issues immediately demanded consideration. These included whether data from OPAL would suffice (and so exclude the 11% of facilities not participating in OPAL), or whether to acquire updated data; whether to count all hospital presentations (including emergency department [ED] visits that did not lead to an admission), or whether to limit rankings to admissions of particular diagnoses. The primary endpoint for ARCHUS was potentially avoidable hospitalisations (PAH). So, should selection be limited to admission within the trial outcome definition, or should all admissions be included – given that at the time of acute referral or presentation the diagnosis may be unclear? Were all care types (high, low, or specialist dementia care) of interest, or should some levels of care be excluded? Should facilities be ranked separately by level of care, or should case-mix be taken into account in some other way? Should analyses be limited to residents in long-stay care (i.e. exclude short-stayers)? What impact would missing data have? What timeframes were relevant? These and other considerations encountered since are structured into Table 1. We suggest these as a framework for others addressing similar problems.

**Table 1:** Selected dimensions when assessing facilities for high use of acute hospitalisations

**Research question: to …**	• Find the fewest facilities to accumulate numbers of hospital events?
	• Identify resident- or facility-level characteristics associated with higher (or lower) event rates so as to inform intervention design?
	• Find facilities that have high hospital presentation rates even if explained by resident characteristics?
	• Find facilities that, independently of their facility or resident characteristics, have high event rates?
	• Find facilities that after adjusting for non-modifiable characteristics, have unexplained high rates of presentations?
**Hospital event type** as endpoint of interest	• All hospital visits, or acute/ED presentations, or acute admissions?
	• All or selected diagnoses only, e.g. those classified as potentially avoidable (PAH)?
	• If only selected diagnoses, e.g. PAH, were codes predefined or selected/amended after data was gathered?
**LTC facility type**	• Limit to particular facility types – e.g. lower-level care?
	• Use only facilities with complete or near-complete data?
	• Is distance or time to hospital likely to impact referral decisions?
	• Use only facilities of a certain size (for power & cost considerations)
	• Need to stratify by e.g. geography, or match in pairs for randomisation?
**Resident care type**	• Use only long-stay residents, or include short-stayers?
	• Limit to those in certain levels of care, e.g. low-level care, or dementia care, or in one age group, or those with public funding, or those with a particular clinical history?
**Cohort assembly**	• Include all residents at any one time, i.e. cross-sectional?
	Or all entering (or leaving) the facility during a pre-defined period?
	Or all using the facility at any time during a period?
**Time period of events**	• Hospital events over what time period?
	• Data collected retrospectively or prospectively?
	• In a special study, or with routine data collection?
**Adjustments during analysis**	• Can results consider person-time, e.g. on death or moving away?
	• Can results consider facility-level characteristics? If so, how?
	• Can results consider resident-level characteristics? If so, how?
**Measure for reporting and ranking**	• Report a count, a proportion, a rate over time, a facility-related effect size from model, a residual from a fitted statistical model, or a change in rank between two methods?
	• Express as rate per bed, per resident, per resident year, or relative to other facilities, to an earlier report or to a “best practice” target?
**Data quality, completeness & recency**	• What is the extent of missingness in data – facilities, outcomes or data items?
	• Is missing data correlated with particular variables so as to lead to bias?
	• Are data current, or could changes have occurred since collection?
	• How reliable are measures, ratings, and coding?

In the years since 2008 when OPAL was conducted, several new facilities had opened, and others had changed ownership or closed. We wished to ensure that all facilities with current certification, regardless of how long in operation, level of care, ownership (part of chain, private or public) should be eligible for selection. It was anticipated that 36 facilities would be recruited for ARCHUS [16]. Investigators determined therefore that a selection process that used OPAL data would be used but would need to be updated using current facility lists and equivalent but more recent data.

### Study outline

The decision was made that we would use data from OPAL, linked to nationally-collected hospitalisation data from 2008–09. The definition of a PAH would match that planned for ARCHUS. Based on statistical models of these resident records and related hospitalisations we would select particular variables that were most predictive of PAHs and that could be updated. New data would then be collected for those variables only, applied at facility-level and facilities ranked accordingly.

### Data sources

OPAL collected data for all residents in 152 of all 172 certified facilities in the region for an estimated 7601 residents [12,17]. Of all certified facilities, 150 provided rest home or lower-level care beds, and 81 provided hospital or higher-level care (59 provided both). Over 98% of residents were long-stay, 71% were women, 91% were aged 65+ years and 34% aged 85+ years [12,17]. Information describing *facility-level characteristics* included structural and process aspects: e.g. size, type (rest home, dementia care, private hospital, psychogeriatric hospital), ownership (for profit or corporate or not-for-profit charitable), levels of nursing and care assistant staffing cover, medical cover, distance from nearest acute hospital, and whether part of a corporate chain or not. *Individual residents’ information* (36 items) included age, ethnicity, length of stay, care level, prior residence, marital status, family contact, function, dependency (mobility, toileting, continence), aspects of cognition, need of night care, ‘problem’ and wandering behaviour, activities of daily living, speech, hearing, vision, medication, recent unplanned GP visits, recent hospital attendance and need for specialized nursing care. Any of these characteristics may be associated with hospitalisations.

Outcomes were obtained from Ministry of Health (MoH) records linked to OPAL data via the National Health Identification number (NHI), NZ’s unique personal health identifier. Historical hospital presentation data included emergency department (ED) presentations and acute unplanned admissions during the two years prior to OPAL. Hospital admission records for the 22 months following OPAL were extracted and all those meeting the definition of potentially avoidable (see below) were selected. Information included diagnoses recorded for discharges following admissions (but not for ED visits not leading to admission), hospital admission date, length of stay and discharge status. Mortality records provided date of death.

Data for hospitalisations within the Auckland region of people in receipt of a residential care subsidy (believed to be about 65% or all LTC residents) during the period January to June 2010, together with summary data for the few predictor variables selected by the model (see below), was obtained from the District Health Boards (DHBs).

### Classification of potentially avoidable hospitalisations

The two closely-allied concepts of PAH and ambulatory sensitive hospitalisations (ASH) have been discussed in the literature. Both PAH and ASH, typically identified by primary diagnosis, are widely accepted in monitoring hospitalisations [18-22]. Purdy et al. have drawn attention to the inconsistent definitions used [23]. Others are developing an international agreed classifications of ASH [19] and PAH [20]. However as yet there are no widely accepted definitions. Two NZ reports focus on people aged under 75 years for whom long-term prevention, for example, is usually justifiable, when it may not be in older people living in LTC settings [18,24].

For this study, we decided that the definition should be purpose-specific and accordingly PAH events was defined as a set of diagnoses broadly congruent with previous studies, but emphasising conditions that related to the ARCHUS trial intervention. Discharges were classified as PAH if the first, second or third diagnosis was one or more of a list including chronic obstructive pulmonary disease, bronchitis, pneumonia, congestive heart failure, dehydration, urinary tract infection, anaemia, cellulitis, leg ulcers, collapse or syncope, constipation, influenza, accidental fractures and other injuries rising from falls, and some less frequently common diagnoses. The ARCHUS list, including detailed ICD-10 codes, is available in Additional file 1 as an online resource.

### Methods of ranking

Facility lists were updated from those used in 2008; (new certified facilities were added, those closed were dropped) to reflect what was current in late 2010. Having determined to use updated data, but without conducting a complete regional survey, data for subsidised residents were assembled from administrative data. Four methods of identifying facilities with high PAH rates were selected for comparison purposes. The first two methods used observed counts of all facilities, the third used predicted counts, and the fourth a combination, but all used updated 2010 data. In all methods, a rank of one signified the lowest PAH use, and a rank of 149 identified the facility ranked highest.

The first (Method 1) was based simply upon the ratio of events to residents, without considering deaths or discharges from the facility. This was used as a comparator for other rankings as it is the simplest and most similar to data available in other settings. This method is likely to be the easiest to replicate in other settings. Secondly, event counts were divided by the sum of resident survival time to derive PAH rates (Method 2) using mean survival time (from OPAL to death) given the resident care level applied to current counts of subsidised residents.

Thirdly, a predictive model was developed that would reduce the number of variables to a few based on what was known about residents currently in the facilities (model-building described below). Data for important variables were updated, converted to facility-level variables (e.g. proportions) and the models applied. Predicted values were the estimated number of events per facility, effectively becoming a predictive risk score for each facility that adjusted for important resident case-mix, and used for ranking in Method 3. Finally, in Method 4 the ranks derived from Method 3 were compared with those observed from Method 2, and ordered by the change in ranks. The intention was to identify how much use each facility was observed above or below what was expected, given facility characteristics and resident case-mix.

### Predictive models

In the first step of statistical modelling, multilevel predictive models were used to account for the hierarchical nature of the data in which residents were nested within facilities, acknowledging that residents within a facility are likely to be somewhat similar [25]. Data were used from residents in all 149 participating facilities that provided linkage information. A generalized mixed model was used to predict event counts for each facility assuming a negative binomial distribution. Facility identifier was entered as a random effect. In all, 146 variables were initially made available to the model, and progressively eliminated if the effect size was closer to zero and the p-value closer to 1. The final model (using information updated from administrative records) included four predictors: residents seen by GP urgently within 2 weeks prior, the proportion of residents seen in ED in the 3-months period 1 year prior, the proportion of residents with a previous history of an admission for diabetes, and the proportion of residents with a previous history of an admission for dementia. All variables retained in the final model had a p-value of 0.05 or under. The model is shown in Additional file 2 (online resource).

In ARCHUS, a method similar to Method 3 was used, but because the trial design necessitated even greater complexity, for delivery and resource reasons stratified by and ranked within DHB (12 facilities from each to provide adequate statistical power).

Analyses were conducted using SAS 9.3 (SAS, Cary, NC). Full ethics approvals for the study were given by the Northern X Regional Ethics Committee (NTX/08/49/EXP and NTX/10/EXP/087).

## Results

**Figure 1:**
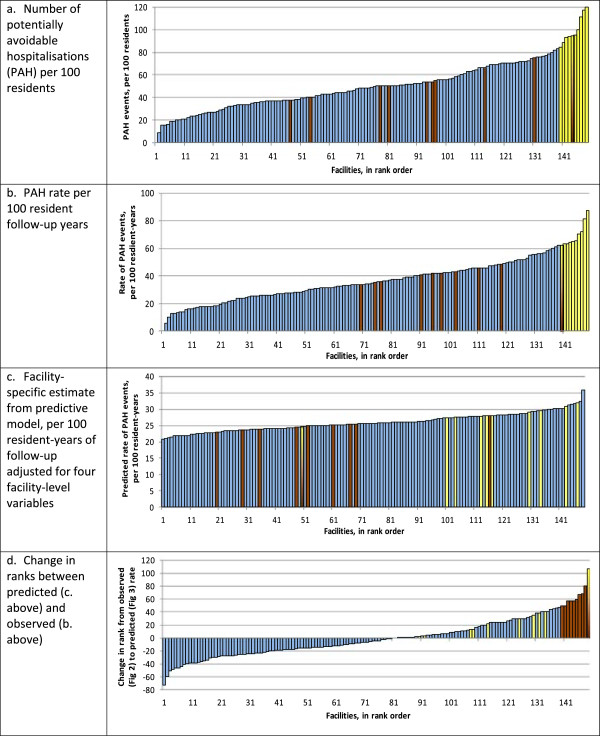
**Comparison of facility rankings by four methods.** Method 1 **(a)** uses simple event counts per person, Method 2 **(b)** event rates per year of resident follow-up, Method 3 **(c)** statistical model of rates using four predictors, and Method 4 **(d)** the change in ranks between methods 2) and 3). The 10 facilities ranked the highest in **a)** are shown in yellow, and the 10 ranked highest in **d)** are shown in brown in all charts, to demonstrate variability between methods. One facility is shown as both yellow and brown.

In all, 3048 PAH events were observed during the 22 months of follow-up following OPAL. The facility-level median PAH event rate in the OPAL cohort was 34 PAH events per 100 person-years of follow-up, and is the best estimate of an overall event rate during the period.Results from all four methods are shown in Figures 1a-d, in which each vertical bar represents one facility. The facility with the lowest PAH event in each method is ranked 1 (left-most), and the highest ranked 149 (right-most) overall across all DHBs. The “top ten” facilities ranked the highest by Method 1, and the “top ten” facilities ranked highest in Method 4, are highlighted in yellow and brown respectively in all charts to visualise the variation between methods. Method 1 was simplest – the ratio of PAH events during the 22 months post-OPAL to 100 residents in that facility in OPAL (Figure 1a). Although simplest, it takes no account of duration of survival, nor of residents leaving during the follow-up period. When so adjusted, as shown in Method 2, few changes in rankings occur (Figure 1b) – all the “top ten” facilities in Method 1 remained in the “top ten” in Method 2.

The statistical model developed in Method 3 found four facility-level predictors as described above. Because the models with OPAL data found that no high-level care facilities had high admission rates, all high-level care facilities were excluded from facility selection. In the model based on OPAL data, all top 20 facilities in each DHB had an observed PAH rate of at last 35 per 100 resident years of follow-up. When updated data were used, the predictive models assessed all top 20-ranked facilities at 31 or more events per 100 years of follow-up. When then ranked by predicted score, one facility highly-listed in earlier rankings became middle-ranked (49^th^) in overall rankings, and the rankings of five others reduced by 18 or more places (Figure 1c), indicating that the higher observed rates in some facilities in Methods 1) and 2) were accounted for by some of the predictors. Using Method 4, the change-in-ranks method (Figure 1d), only one facility previously identified as highly-ranked remained highly-ranked.

## Discussion

This study demonstrates a few possible methods for selecting LTC facilities with high use of acute hospital admissions, ranging from simpler methods to complex statistical models. Rankings varied markedly. Use of one method without considering other options may well have selected facilities in which the trial intervention would have been irrelevant, defeating the purpose of running a selection process. No method identified a group with clearly higher use – the figures indicate a continuum of risk, not any obvious distinction between facilities with higher or lower use.

This comparison of methods shows that for ARCHUS, a simple assessment of rate of admission given facility size would not have adequately selected facilities that should fairly be regarded as high users of acute hospital services. To illustrate how different the methods were, one facility (with progressive shading in the plots) ranked in the top ten in Methods 1, 2 and 4, but ranked only 50^th^ of 149 in Method 3, indicating that its predicted need was not high. In others this change did not occur: two facilities ranked in the top ten by three methods (1, 2 and 3), suggesting that although rates in these facilities were high, they were expected to be so given the casemix of their residents. The highest-ranked facility in Method 3 was ranked outside the top ten (129^th^ and 132^nd^) in Methods 1 and 2, suggesting that although the rate of PAH admissions was not high, resident care needs were relatively low and PAH rates were higher than expected.

Statistical approaches to provider profiling and risk adjustment have been previously discussed. Berlowitz et al. showed that using Bayesian methods reduced numbers of outlying LTC facilities by adjusting not to the norm but to clinical best practice [26]. Roy and Mor drew attention to measurement of quality and the importance of unbiased coding in data collection [27]. Li et al. showed the importance of adjusting for individual and facility-level characteristics when describing nursing home care quality [28]. Austin and Reeves, in a study assessing models of hospital care quality, concluded that the c-statistic was of little use to assess model fit although widely-accepted [29]. Ieva and Paganoni successfully used mixed effect models and funnel plots in hospital readmission rates [30]. Most recently, Eijkenaar and van Vliet compared rankings of quality, not of LTC facilities but of primary care providers, using many different statistical models to identify outliers [31]. They observed similar results from the models but very varied outliers and rankings, notably that the models better detected *high*-performing providers than *low*-performing providers. They too emphasised the care needed in selecting the model and noted variability over time. All these methods assume the availability of good, recent data, such as are available in jurisdictions such as the USA where NMDS data and other large datasets exist. In other settings such as NZ, reusing available data presents additional challenges.

Less discussion has centred on non-statistical aspects. In the ARCHUS trial, the aim was to select and target high-use facilities in order to provide a multi-disciplinary intervention comprising staff education, clinical supports, and medical and pharmacologic reviews of residents. A model that included admissions over a prior period as well as what was known about current (albeit subsidised) residents was used. Detailed results of models and rankings are not central to this paper; the intent is to identify more general issues and options, analytical and otherwise, that warrant consideration in determining appropriate methods. In hindsight, the ARCHUS team may have done things differently – for example we did not anticipate that non-participation would be as high as it was. The concept of selecting only high-use facilities, once the number had been reduced by removing high-level care facilities, and some declined to participate, meant that to recruit 36 facilities, 12 in each DHB region, some mid-ranked facilities had to be recruited. The concept of outliers as discussed by Ieva and Paganoni [30] indicates that selecting high-use facilities is feasible only if numbers of facilities are sufficient that outliers can realistically be identified, not when smaller numbers are available. But even then, the work of Eijkenaar and van Vliet [31] suggests that modelling low-outliers rather than high-outliers may have achieved greater discrimination.

The variations in rankings derived under different methods show the need for careful attention, in particular to the purpose of the question. Had we wished simply to identify facilities with *highest numbers* of hospital events (e.g. to monitor performance during a period of service change, regardless of resident needs), then a recent simple rate, count per resident, or count per bed, may have sufficed. Had we wished to *predict high counts* (e.g. to recruit the fewest possible facilities to most quickly accumulate events for a research project while minimising travel and overhead costs), then use of recent history of events is highly predictive, though it takes no account of whether the high rates are due to resident needs or case-mix within the facilities, to quality of care or to other factors such as distance from hospital that may impact on the decision to send a resident to hospital. Had we wished to *identify characteristics* that may lead to high expenditure on hospital events to inform service design, statistical models to identify factors most associated with costs of event are more appropriate. In that case, unlike when good predictive ability is required, measures of actual recent observed events would probably not be considered in the model in order not to perpetuate existing patterns but to allow them to be identified.

However, when the purpose is to *identify and target* high-use facilities where changes may be achievable, for example to benchmark quality of care more robustly, or to trial an intervention (as was intended in ARCHUS), it is preferable to select facilities with higher-than-expected rates i.e. higher than predicted after adjustment for underlying facility-level risk factors and/or resident case-mix, without including observed rates for an earlier period. In such cases, if current and reliable data is available, then differences between predicted (viz. modelled) rates and observed rates (i.e. model residuals) would have been preferred. The combinations of the issues listed in Table 1 are many but each issue should be explicitly considered.

Definitional issues of PAH events have been raised above and elsewhere. It is possible that simply using the first, or first three, diagnosis codes does not adequately take into account complex cares such as typically occurs in multi-morbid presentations of frail older people. One option is to clinically review all medical records in detail, but that is extremely resource intensive and subject to personal opinion. Even then, review is unlikely to correctly classify all cases as “avoidable” or “unavoidable”; data limitations are unlikely for example to describe competing demands being placed upon facility staff at the time of an episode needing greater care and which may clinch the decision to hospitalise. Regardless, the definition itself is not central to his report, though having a definition is.

## Conclusions

Countries including NZ have strategies to reduce LTC placement by supporting ‘ageing in place’ [32]. However there will always be a need for LTC as the scope for home care for the highly dependent is limited [33]. It is important to ‘better manage’ LTC care to improve quality [34] and to use funding efficiently. With the healthcare workforce in short supply [35,36], new ways of working are called for, to better support the industry and improve resident outcomes, for example by reducing acute hospitalisations. If an intervention designed to reduce acute referrals to hospital (assuming reasonable preventive care has been undertaken) by providing some hospital-level care within the LTC facility, then all acute presentations are likely to be the outcome of interest. Where an intervention focuses on preventive care to reduce admissions from consequences of care over a longer period, such as avoiding pressure sores, then a model predicting all hospital presentations (including non-acute admissions) with appropriate discharge diagnoses may be more relevant, with recognition given to underlying need levels of residents. In some instances, acute hospital referrals managed within an ED, and not admitted, may be as relevant, or more, than admissions themselves.

This paper offers some considerations when making choices about methods. Not all dimensions shown in Table 1 will be meaningful for all occasions, but a review of them may avert needless effort and rework, and provide clarity earlier in the selection process.

## Abbreviations

ARCHUS: Aged residential care healthcare utilization study; ASH: ambulatory sensitive hospitalisations; ED: Emergency department; LTC: Long-term care; MoH: Ministry of Health; NHI: National Health *i*ndex; NMDS: National Minimum Data Set; NZ: New Zealand; OPAL: Older people’s ability level; PAH: Potentially avoidable hospitalisations.

## Competing interests

The authors declare they have no competing interests.

## Authors’ contributions

JBB conceived the research question, designed and conducted all analyses and prepared and revised drafts. TA and TL supervised the work. MJC provided support. MB & NK and all authors provided critical review and comment. All authors read and approved the final manuscript.

## Pre-publication history

The pre-publication history for this paper can be accessed here:

http://www.biomedcentral.com/1471-2288/14/93/prepub

## Supplementary Material

Additional file 1 Table of classification of PAH events using ICD codes of first three diagnoses. Click here for file

Additional file 2 Model used in ARCHUS to select facilities with high risk of PAH*. Click here for file

## References

[B1] Dosa D McNicoll L Infections in the nursing home: a primer for the practicing physician Med Health R I 2007 90 7 211 212 216–217 17711079

[B2] Fried TR Mor V Frailty and hospitalization of long-term stay nursing home residents J Am Geriatr Soc 1997 45 265 269 9063269 10.1111/j.1532-5415.1997.tb00938.x

[B3] Gillick M Steel K Referral of patients from long-term to acute-care facilities J Am Geriatr Soc 1983 31 2 74 78 6822711 10.1111/j.1532-5415.1983.tb05418.x

[B4] Rubenstein LZ Ouslander JG Wieland D Dynamics and clinical implications of the nursing home-hospital interface Clin Geriatr Med 1988 4 471 491 3044556

[B5] Gill TM Allore HG Holford TR Guo Z Hospitalization, restricted activity, and the development of disability among older persons JAMA 2004 292 17 2115 2124 10.1001/jama.292.17.2115 15523072

[B6] Steel K Gertman PM Crescenzi C Anderson J Iatrogenic illness on a general medical service at a university hospital New Engl J Med 1981 304 11 638 642 10.1056/NEJM198103123041104 7453741

[B7] Ouslander JG Reducing the hospitalization of nursing home residents J Am Geriatr Soc 1988 36 2 171 173 3339221 10.1111/j.1532-5415.1988.tb01788.x

[B8] Bo M Martini B Ruatta C Massaia M Ricauda NA Varetto A Astengo M Torta R Geriatric ward hospitalization reduced incidence delirium among older medical inpatients Am J Geriatr Psychiatry 2009 17 9 760 768 10.1097/JGP.0b013e3181a315d5 19705520

[B9] Vohra JU Brazil K Hanna S Abelson J Family perceptions of end-of-life care in long-term care facilities J Palliat Care 2004 20 4 297 302 15690832

[B10] Boockvar KS Gruber-Baldini AL Burton L Zimmerman S May C Magaziner J Outcomes of infection in nursing home residents with and without early hospital transfer J Am Geriatr Soc 2005 53 4 590 596 10.1111/j.1532-5415.2005.53205.x 15817003

[B11] Boockvar KS Gruber-Baldini AL Stuart B Zimmerman S Magaziner J Medicare expenditures for nursing home residents triaged to nursing home or hospital for acute infection J Am Geriatr Soc 2008 56 7 1206 1212 10.1111/j.1532-5415.2008.01748.x 18482299 PMC3766964

[B12] Broad JB Boyd M Kerse N Whitehead N Chelimo C Lay-Yee R von Randow M Foster S Connolly MJ Residential aged care in Auckland, New Zealand 1988–2008: do real trends over time match predictions? Age Ageing 2011 40 4 487 494 10.1093/ageing/afr056 21628389

[B13] Barker WH Zimmer JG Hall WJ Rate, patterns, causes and costs of hospitalization of nursing home residents: a population-based study Am J Public Health 1994 84 1615 1620 10.2105/AJPH.84.10.1615 7943480 PMC1615106

[B14] Freiman MP Murtaugh CM Interactions between hospital and nursing home use Public Health Rep 1995 110 546 564 7480608 PMC1381627

[B15] Ingarfield SL Finn JC Jacobs IG Gibson NP Holman CD Jelinek GA Flicker L Use of emergency departments by older people from residential care: a population based study Age Ageing 2009 38 3 314 318 19286676 10.1093/ageing/afp022

[B16] Foster SJ Boyd M Broad JB Whitehead N Kerse N Lumley T Connolly MJ Aged residential care health utilisation study (ARCHUS): a randomised controlled trial to reduce acute hospitalisations from residential aged care BMC Geriatr 2012 12 54 doi: 10.1186/1471-2318-12-54 10.1186/1471-2318-12-54 PMC3489701 22974314

[B17] Boyd M Broad JB Kerse N Foster S von Randow M Lay-Yee R Chelimo C Whitehead N Connolly MJ Twenty-year trends in dependency in residential aged care in Auckland, New Zealand: a descriptive study J Am Med Dir Assoc 2011 12 7 535 540 10.1016/j.jamda.2011.01.014 21450250

[B18] Jackson G Tobias M Potentially avoidable hospitalisations in New Zealand, 1989–98 Aust N Z J Public Health 2001 25 3 212 221 10.1111/j.1467-842X.2001.tb00565.x 11494988

[B19] Walker J Teare G Hogan D Lewis S Maxwell C Identifying potentially avoidable hospital admissions from Canadian long-term care facilities Med Care 2009 47 2 250 254 10.1097/MLR.0b013e3181847588 19169127

[B20] Ouslander JG Maslow K Geriatrics and the triple aim: defining preventable hospitalizations in the long-term care population JAMA 2012 60 12 2313 2318 10.1111/jgs.12002 23194066

[B21] Billings J Zeitel L Lukomnik J Carey TS Blank AE Newman L Impact of socioeconomic status on hospital use in New York City Health Aff (Millwood) 1993 12 1 162 173 10.1377/hlthaff.12.1.162 8509018

[B22] Blunt I Focus on preventable admissions: trendds in emergency admissions for ambulatory care sensitive conditions, 2001 to 2013 2013 London: The Health Foundation and the National Trust

[B23] Purdy S Griffin T Salisbury C Sharp D Ambulatory care sensitive conditions: terminology and disease coding need to be more specific to aid policy makers and clinicians Public Health 2009 123 2 169 173 10.1016/j.puhe.2008.11.001 19144363

[B24] Waitemata District Health Board Waitemata health needs assessment 2009: health and health care of Waitemata residents 2009 Waitemata District Health Board: North Shore

[B25] Harrell FE Regression modeling strategies: with applications to linear models, logistic regression, and survival analysis 2007 New York: Springer

[B26] Berlowitz DR Christiansen CL Brandeis GH Ash AS Kader B Morris JN Moskowitz MA Profiling nursing homes using Bayesian hierarchical modeling J Am Geriatr Soc 2002 50 6 1126 1130 10.1046/j.1532-5415.2002.50272.x 12110077

[B27] Roy J Mor V The effect of provider-level ascertainment bias on profiling nursing homes Stat Med 2005 24 23 3609 3629 10.1002/sim.2215 16158404

[B28] Li Y Cai X Glance LG Spector WD Mukamel DB National release of the nursing home quality report cards: implications of statistical methodology for risk adjustment Health Serv Res 2009 44 1 79 102 10.1111/j.1475-6773.2008.00910.x 19146565 PMC2669625

[B29] Austin PC Reeves MJ The relationship between the C-statistic of a risk-adjustment model and the accuracy of hospital report cards: a Monte Carlo Study Med Care 2013 51 3 275 284 10.1097/MLR.0b013e31827ff0dc 23295579 PMC4617826

[B30] Ieva F Paganoni AM Detecting and visualizing outliers in provider profiling via funnel plots and mixed effect models Health Care Manag Sci 2014 doi: 10.1007/s10729-013-9264-9 10.1007/s10729-013-9264-9 24402171

[B31] Eijkenaar F van Vliet RC Performance profiling in primary care: does the choice of statistical model matter? Med Decis Making 2014 34 2 192 205 10.1177/0272989X13498825 23920433

[B32] Ministry of Health Health of older people strategy 2002 Wellington: Ministry of Health

[B33] Weatherall M Slow M Wiltshire K Risk factors for entry into residential care after a support needs assessment N Z Med J 2004 117 1202 1075 15477909

[B34] Flicker L Healthcare for older people in residential care-who cares? Med J Aust 2000 173 2 77 79 10937032 10.5694/j.1326-5377.2000.tb139247.x

[B35] Zurn P Dumont J-C Health workforce and international migration: can New Zealand compete? 2008 Paris: OECD

[B36] Cox M Hope S Davies P for NZIER (New Zealand Institute of Economic Research) Ageing New Zealand and health and disability services: demand projections and workforce implications, 2001–2021: a discussion document 2004 Wellington: Ministry of Health

