# Supplementary material for: Selecting long-term care facilities with high use of acute hospitalisations: issues and options
Source: BMC Med Res Methodol. 2014 Jul 22;14:93. doi: 10.1186/1471-2288-14-93 (PMC4118262; doi:10.1186/1471-2288-14-93)
Supplement: Additional file 1 — Table of classification of PAH events using ICD codes of first three diagnoses. [file 1471-2288-14-93-S1.docx]

## Additional file 1: Table of classification of PAH events using ICD codes of first three diagnoses

| **Conditions included** | **Diagnosis Codes (ICD-10)   - except Falls based on Injury Code** |
| --- | --- |
| Cellulitis | H000, H010, J340, L01-L04, L08, L980 |
| Chronic obstructive pulmonary disease (COPD) & asthma | J20-J22, J44-J46 |
| Collapse or syncope | R55 |
| Congestive heart failure (CHF) | I50, J81 |
| Constipation | K590 |
| Dehydration, weight loss, gastroenteritis, volume depletion | A02-A09, R11, E86, E87, T73 |
| Fractures  (if fracture date was not after admission date) | S02, S04, S12, S22, S32, S42, S52, S62, S72, S82, S92, T02, T08, T10, T12 |
| Other injuries - many types  (if injury date was not after admission date) | S03, S06, S13, S14, S20, S23, S24, S27, S30, S33, S34, S40, S41, S43, S44, S50, S51, S53, S54, S60, S61, S63, S64, S67, S70, S71, S73, S74, S80, S81, S83, S84, S90, S91, S93, S94, T01, T03, T09, T11, T13, T14 |
| Falls (if not fracture or other injury and  if fall date was not after admission date) | Injury code is W01, W03, W07-W08, W10, W13, or W17-W19 |
| Nutrition deficiency and anaemia | D50-D53, E40-E46, E50-E64, M833 |
| Pneumonia, influenza, bronchitis, pneumonitis & other lower respiratory tract infections | J10-J16, J18, J69 |
| Skin tears, lacerations, pressure ulcers, leg ulcers | I83, L89, L97, L984, T14 |
| Urinary tract (UTI) or kidney infections | N10, N12, N136, N309, N390 |
| Angina and chest pain | I20, R072-R074 |
| Dementia, or Behavioural and Psychological symptoms of Dementia (BPSD) & related problems | F00, F01, F03, F05, F060, F063, F064, F067, F069, F070, F09, G30, G312, G318 |
| Depression | F32, F33, F34, F38 |
| Diabetes & diabetes-related complications | E10-E14, E162 |
| Hypotension | I95 |
| Pain | F454, M25, M79, R52 |
| Paralytic ileus & obstruction | K56 |
| Poisoning, allergic response, & ADRs | T36-T50, T78, Y40-Y84 |
| Sepsis | A40, A41, A49, B25 |
| Tube block: catheter, PEG or naso-gastric | T830-T832, T834-T839 |
| Upper respiratory tract (URTI) & ENT infections | J00-J04, J06, H65-H67 |
