# Supplementary material for: Selecting long-term care facilities with high use of acute hospitalisations: issues and options
Source: BMC Med Res Methodol. 2014 Jul 22;14:93. doi: 10.1186/1471-2288-14-93 (PMC4118262; doi:10.1186/1471-2288-14-93)
Supplement: Additional file 2 — Model used in ARCHUS to select facilities with high risk of PAH*. [file 1471-2288-14-93-S2.docx]

## Additional file 2: Model used in ARCHUS to select facilities with high risk of PAH*

Rate ratio 95%CI

Resident seen by GP urgently within 2 weeks
prior to OPAL (Y vs. N) 1.79 1.55 to 2.06

Proportion of residents seen in ED in the 3-months
period 1year prior (each 10%) 1.14 1.07 to 1.20

Proportion of residents with a previous history
of an admission for diabetes (each 10%) 1.17 1.00 to 1.36

Proportion of residents with a previous history
of an admission for dementia (each 10%) 1.10 0.99 to 1.22

*PAH = potentially avoidable admissions
